# Tribological and Wear Performance of Nanocomposite PVD Hard Coatings Deposited on Aluminum Die Casting Tool

**DOI:** 10.3390/ma11030358

**Published:** 2018-02-28

**Authors:** Jose Mario Paiva, German Fox-Rabinovich, Edinei Locks Junior, Pietro Stolf, Yassmin Seid Ahmed, Marcelo Matos Martins, Carlos Bork, Stephen Veldhuis

**Affiliations:** 1 McMaster Manufacturing Research Institute (MMRI), Department of Mechanical Engineering, McMaster University, 1280 Main Street West, Hamilton, ON L8S4L7, Canada; gfox@mcmaster.ca (G.F.-R.); lockse@mcmaster.ca (E.L.J.); stolfp@mcmaster.ca (P.S.); seidahmy@mcmaster.ca (Y.S.A.); carlosbork@gmail.com (C.B.); veldhu@mcmaster.ca (S.V.); 2 Department of Mechanical and Materials Science, Catholic University of Santa Catarina, Rua Visconde de Taunay, 427-Centro, Joinville, SC 89203-005, Brazil; marcelo.martins@catolicasc.org.br; 3 IFSul—Federal Institute Sul-rio-grandense—Campus Sapucaia do Sul, Av Copacabana, 100, Sapucaia do Sul, RS 93216-120, Brazil

**Keywords:** PVD nanocomposite coatings, aluminum die casting, tool life, tribological performance

## Abstract

In the aluminum die casting process, erosion, corrosion, soldering, and die sticking have a significant influence on tool life and product quality. A number of coatings such as TiN, CrN, and (Cr,Al)N deposited by physical vapor deposition (PVD) have been employed to act as protective coatings due to their high hardness and chemical stability. In this study, the wear performance of two nanocomposite AlTiN and AlCrN coatings with different structures were evaluated. These coatings were deposited on aluminum die casting mold tool substrates (AISI H13 hot work steel) by PVD using pulsed cathodic arc evaporation, equipped with three lateral arc-rotating cathodes (LARC) and one central rotating cathode (CERC). The research was performed in two stages: in the first stage, the outlined coatings were characterized regarding their chemical composition, morphology, and structure using glow discharge optical emission spectroscopy (GDOES), scanning electron microscopy (SEM), and X-ray diffraction (XRD), respectively. Surface morphology and mechanical properties were evaluated by atomic force microscopy (AFM) and nanoindentation. The coating adhesion was studied using Mersedes test and scratch testing. During the second stage, industrial tests were carried out for coated die casting molds. In parallel, tribological tests were also performed in order to determine if a correlation between laboratory and industrial tests can be drawn. All of the results were compared with a benchmark monolayer AlCrN coating. The data obtained show that the best performance was achieved for the AlCrN/Si_3_N_4_ nanocomposite coating that displays an optimum combination of hardness, adhesion, soldering behavior, oxidation resistance, and stress state. These characteristics are essential for improving the die mold service life. Therefore, this coating emerges as a novelty to be used to protect aluminum die casting molds.

## 1. Introduction

Aluminum is a widely used material in the automotive industry. The use of special aluminum alloys as materials to manufacture components and automotive parts allows for the construction of light-weight components that lead to an overall weight reduction, and thus, to reduced fuel consumption [1]. These aluminum alloys are commonly cast using the high pressure die casting (HPDC) process, which is one of the most efficient methods for the production of complex shape castings in today’s manufacturing industry [2]. For that reason, recent research in this field has focused to improve tool life of aluminum die casting molds.

During the casting of aluminum alloys, the cyclic process leads to thermocyclic loads on the tool surface from T = 90 °C at cooling up to T = 600 °C at die casting. In this way, the tools are exposed to erosion, corrosion, soldering, or die sticking due to the frequent contact between the tool surface and the casting alloy. Any one of these phenomena can result in damage to the die and poor surface quality of the casting, as well as a notable decrease in the productivity and efficiency of the casting operation [3].

In order to protect die casting molds, hard coatings deposited by physical vapor deposition (PVD), such as TiN, CrN, (Cr,Al)N, Ti(C,N), Ti(B,N), or (Ti,Al)(C,N) [4,5,6] have been employed to act as a physical barrier to the die casting molding to prevent the erosion and soldering of aluminum and improve the resistance against thermal cracking [7]. Previous studies show that TiN presented good corrosion and erosion wear resistance [4,5,6]. However, this coating is not a good solution for HPDC due to its low oxidation temperature [4]. TiAlN and TiSiN coatings exhibit good mechanical properties and also exhibit a better oxidation resistance up to 700 °C, however, their adhesion under substrate is reduced, which leads to sudden failures [8] during service. Thus, novel solutions need to be studied in order to reduce the intensity of frictional interaction between the tool surface and the aluminum casting alloy and reduce wear of the molds. 

The coatings previously studied do not show properties good enough to solve the problems outlined above. Therefore, the development of advanced PVD hard coatings, such as nanocomposites, which are designed to resist under severe mechanical and thermal stress conditions emerge as possible solutions to drastically reduce outlined problems. This family of coatings has a nano-crystalline structure that results in high mechanical and functional properties. The coatings of such structure are able to maintain a low friction coefficient (self-lubricating coatings) in extreme environments, combined with high hardness to improve the mechanical resistance [9,10]. Furthermore, the adhesion strength for nanocomposite coating at the interface between the coating and substrate is improved in comparison to regular coatings, and it can guarantee desirable surface properties, as well as long durability of the tool [11]. 

When considering the different characteristics of the nanocomposite coatings during service, the goal of this paper is to present the nanocomposite (AlTiN/Si_3_N_4_ and AlCrN/Si_3_N_4_) coatings that have been deposited by PVD as novel coatings for the aluminum die casting process. To demonstrate suitability, studies of the mechanical properties and tribological performance have been investigated in both laboratory and industrial tests. The results that were obtained in terms of tribological and wear performance were compared with a monolayer AlCrN, which is the coating currently used in industry.

## 2. Materials and Methods

Coatings were deposited onto (AISI H13) hot worked tool steel substrate (48 ± 1 HRC) using a physical vapor deposition (PVD) process. The chemical composition of AISI H13 was provided by the supplier, and it is presented in Table 1.

**Figure 1:**
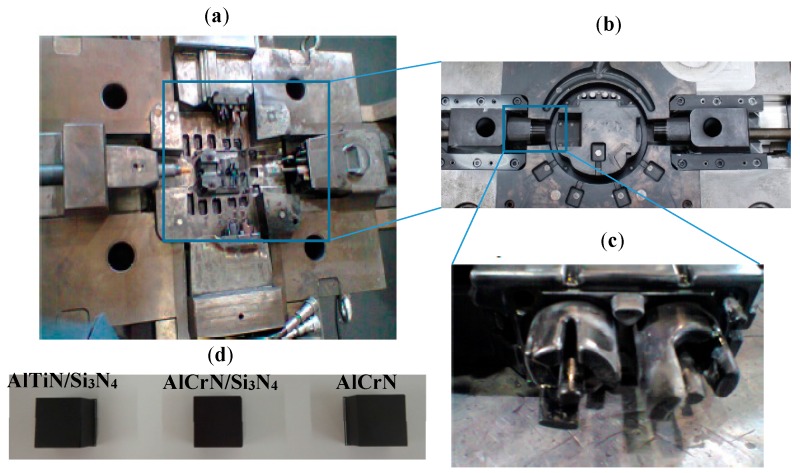
Set of coated samples (**a**) General view from the coated mold; (**b**) cavity details; (**c**) core pins; and (**d**) H13 blocks used for coating characterization studies.

One set of samples (die casting mold, core pins, and blocks) were used for each coating. Figure 1 shows the samples that were used for coating characterization and tool life tests.

The PVD nanocomposites (AlTiN/Si_3_N_4_ and AlCrN/Si_3_N_4_), and AlCrN coatings were deposited on the samples by means of cathodic arc evaporation in a Platit π411 industrial deposition unit, equipped with three lateral arc-rotating cathodes (LARC) and one central rotating cathode (CERC). Plansee, AG—Germany produced all of the targets that were employed in this work. The target that was used for AlCrN coating contained Al/Cr (Al60:Cr40 at %). The deposition process was carried out at a temperature of 500 °C in a 99.999% pure nitrogen atmosphere at a pressure of 4 Pa and a bias voltage of −40 V. To produce nanocomposite coatings (AlTiN/Si_3_N_4_ and AlCrN/Si_3_N_4_), targets containing pure metals (Cr, Ti) and the AlSi (88:12 at %) were employed. The coatings were deposited at a temperature of 480 °C, and two different gases, argon and nitrogen (both 99.999% pure), were used. The pressure of the process was about 3.5 to 4 Pa and the bias voltage of −40 V. During the process, evaporated metals and metal alloys enter the plasma state to combine with the ionized process gas (nitrogen) and eventually condense on the substrate surface, as part of ceramic compounds. Amorphous and micro-nanocrystalline structures and layers are developed with optimized thermodynamic and kinetic conditions. Spinoidal decomposition allows for building TiN and CrN nanocrystalline structures dispersed in a Si_3_N_4_ amorphous matrix, with a typical crystallite size of about 10 nm [12]. 

The roughness of the substrate and coating surfaces were determined by atomic force microscopy (AFM) equipped with a scanning probe (Shimadzu SPM 9500 J3, Kyoto, Japan). A single crystal silicon with a long rectangular cantilever was used as a scanning probe. The tips are pyramidal shaped, with a nominal radius of 10 nm with a spring constant of 0.5 N/m. The scanning mode was configured as contact, with a scanning rate of 1 Hz and high resolution. A region with the size of 30 μm × 30 μm was selected for the characterization of the coated surface samples. The images that were produced were processed to remove background signals, and to extract results such as surface roughness (Ra) and topographic profiles. 

The cross-sections of the coatings were inspected with a field emission gun microscope Supra 55VP by Zeiss (Oberkochen, Germany), equipped with energy-dispersive X-ray spectroscopy (EDS) Quantax XFlash 6/30 by Bruker (Berlin, Germany). The elemental compositions of the nanocomposite coatings were determined by glow discharge optical emission spectroscopy (GDOES) GDS-850A by Leco (Saint Joseph, MI, USA). Five points were analyzed for each sample that was coated, and the average result was taken. The phases of the coatings and residual stress were characterized using X-ray diffraction XDR 7000 by Shimadzu (Kyoto, Japan) with 0.5° grazing angle, scanning range from 30° to 70°, the angle of incidence of 0.02°, and scanning speed of 1°/min. 

Hardness (H) and elastic modulus (E) of the coatings were measured using a G200 XP-MTS Nano Indenter System (Agilent, Boston, MA, USA) equipped with a Berkovich Indenter. To determine the Hardness (H) and elastic modulus (E), the load of 400 mN was applied through 25 indentations that were arranged in a matrix of 5 × 5. To obtain a reliable mean value and standard deviation, at least six points were tested for each sample. Table 2 shows mechanical properties, residual stress, and the thickness of the coatings that were investigated in this work.

Indentation adhesion tests evaluated the quality of the adhesion between the coating and substrate. These tests were carried out on the Wilson instruments Rockwell hardness tester (Buehler, Norwood, UK) at an indentation load of 1471 N (150 kgf) was performed to assess the quality of the coatings. The test procedure followed the VDI 3198 (1991) standard [13]. In addition, a CSM Instruments Revetest Scratch Tester (Anton-Paar, Buch, Switzerland) equipped with a diamond cone (radius of 200 μm, cone angle of 120°) was employed to determine the adhesion of the coatings on the H13 blocks. A progressive normal load, ranging from 1 to 200 N, was applied over a length of 3 mm. An average of three scratch tests was carried out for each coating.

Tribological tests were performed on a pin-on-disk CSM Tribometer (Anton-Paar, Buch, Switzerland) to determine the friction behavior of the coatings at different ranges of temperature. The tests were performed at room and elevated temperature (200, 400 and 600 °C) with a constant load of 20 N on the pin (WC ball was used as a pin), the sliding speed of 0.2 m/s and slide distance of 300 m. The pin-on-disk experiments were repeated at least three times for each temperature to check the reproducibility of the test results. Based on that, the scatter of the friction coefficient values was found to be approximately 5%. The three-dimensional surface profile and depth of the friction zone was assessed using a non-contact Optical Surface Profilometer Zygo-6000 Series (ZYGO, Middlefield, OH, USA).

To evaluate the performance of the coatings, coated mold and core pins were tested during the industrial process using an industrial pressure die casting of the Aluminum alloy AlSi_12_Cu_3_. The casting conditions and the spraying of the lubricating compound were not changed from the standard procedures. The melt temperature in the holding furnace was maintained at 680 °C throughout the experiments. The die gate velocity was 60 m/s, and a final intensification pressure of approximately 65 MPa was applied. The tool life results were measured in terms of parts and hours of production. After the operation, the cores were examined using SEM microscopy (ZEISS, Oberkochen, Germany).

## 3. Results and Discussion

### 3.1. Coatings Characterizations

**Figure 2:**
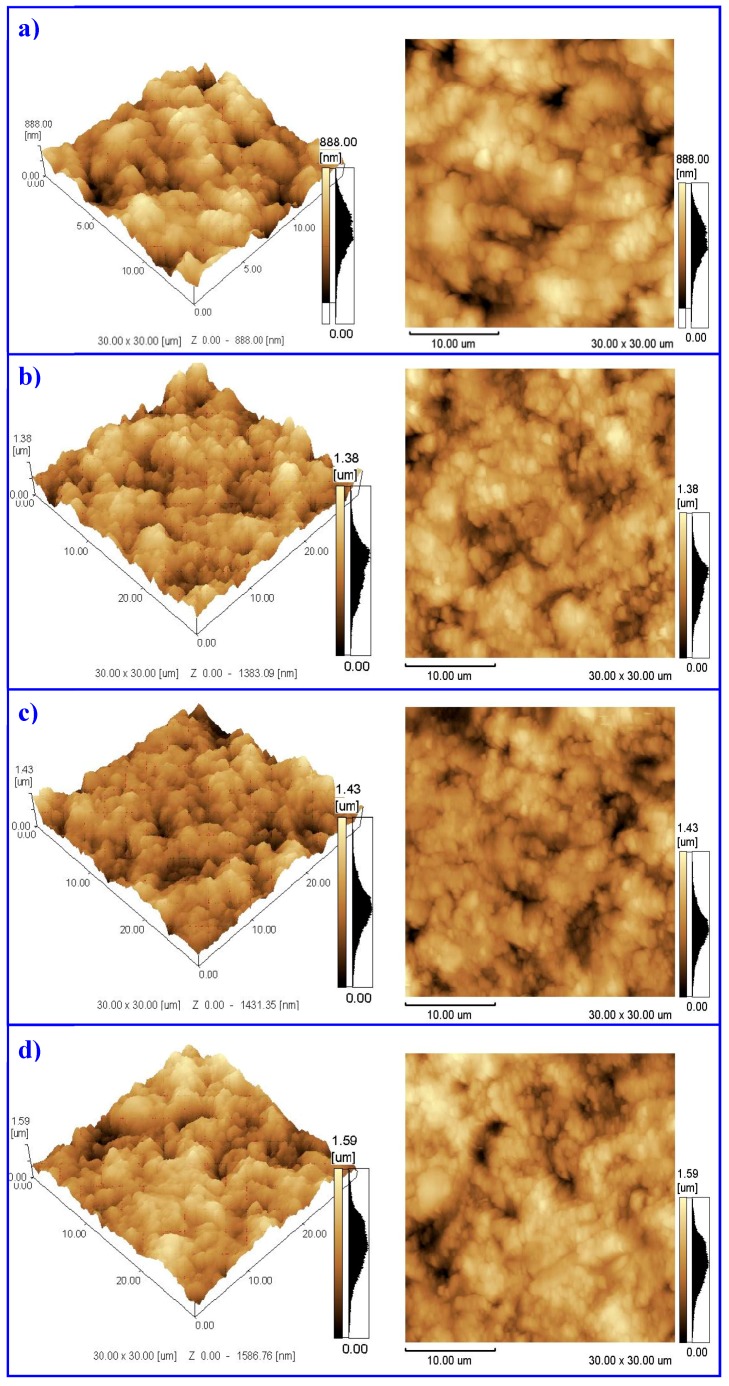
Atomic force microscope images of surface topography. (**a**) uncoated sample; (**b**) AlCrN/Si_3_N_4_ physical vapor deposition (PVD) nanocomposite coating; (**c**) AlTiN/Si_3_N_4_ PVD nanocomposite coating and (**d**) AlCrN PVD Coating.

The morphology of the surfaces was evaluated by Atomic Force Microscopy (AFM). The substrate and coatings’ roughness values obtained in three-dimensional (3D) and two-dimensional (2D) models are presented in Figure 2.

As it is shown, coatings present higher roughness values compared to the substrate, which can be confirmed through of surface topographies analyzed by AFM (Figure 2a). As a final result after the coating deposition process, the lowest surface roughness Ra was observed in AlCrN/Si_3_N_4_ coating (Table 3), and then, confirmed by AFM images (Figure 2b). A summary of the average and standard deviation values of the surface roughness are presented in Table 3.

From the data presented in Figure 2 and Table 3, we can confirm an increase in surface roughness compared to the uncoated state. Nanocomposite hard coatings with increased AlSi deposited by PVD using the Lateral Rotating Cathode (LARC) system allows for obtaining an increase of Ra surface roughness if compared to the substrate. This is due to the lower melting point of the evaporated material during cathodic arc deposition [14]. Increasing cathode current leads to the emission of a higher quantity of particles with a bigger volume and size. As a final result, it is expected that the amount of cathode material would rise with a lower melting point, which would then lead to an increase in the surface roughness [14]. Even though a quick increasing in Ra has been seen, the cathodic arc evaporation process using LARC technology is able to produce very smooth surface roughness when compared to conventional cathodic arc evaporation process [15]. However, in this way, one realizes that nanocomposite coatings present low roughness than AlCrN coating.

**Figure 3:**
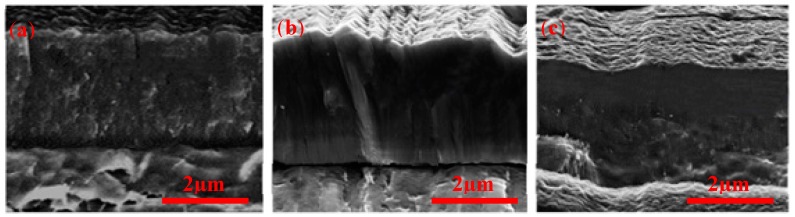
Scanning electron microscopy (SEM) images of coatings cross-section and glow discharge optical emission spectroscopy (GDOES) results for coating chemical composition, (**a**) AlCrN PVD Coating; (**b**) AlTiN/Si_3_N_4_ PVD nanocomposite; and (**c**) AlCrN/Si_3_N_4_ PVD nanocomposite coating.

Figure 3 shows SEM cross-section images for H13 blocks coated with AlCrN, AlTiN/Si_3_N_4_, and AlCrN/Si_3_N_4_. The coatings present a compact structure, without any visible delaminations or defects.

A dense and uniform structure characterizes the morphology of the fracture of coatings studied (Figure 3). AlCrN and AlTiN/Si_3_N_4_ coatings present a columnar structure, while AlCrN/Si_3_N_4_ coating, a randomized, the nearly flawless structure can be observed. Furthermore, the interface of AlCrN/Si_3_N_4_ and the H13 substrate shows no irregularities. However, for AlCrN/Si_3_N_4_ coating, it is apparent that, especially in the lower part of the PVD layer, a slight orientation in the growth direction is existent. Furthermore, the coating gets finer with a decreasing orientation and becoming an amorphous structure as dropless. This observation is supported by the presence of higher content of Silicon at the structure. The GDOES results for the chemical composition of the coatings are shown in Figure 3, as well. The results indicate that the amount of Al content (at %) for AlCrN/Si_3_N_4_ nanocomposite coatings is higher than for AlTiN/Si_3_N_4_. This higher Al content can increase the hardness and the temperature oxidation resistance [16], and may partly explain the better performance for coated die casting. 

**Figure 4:**
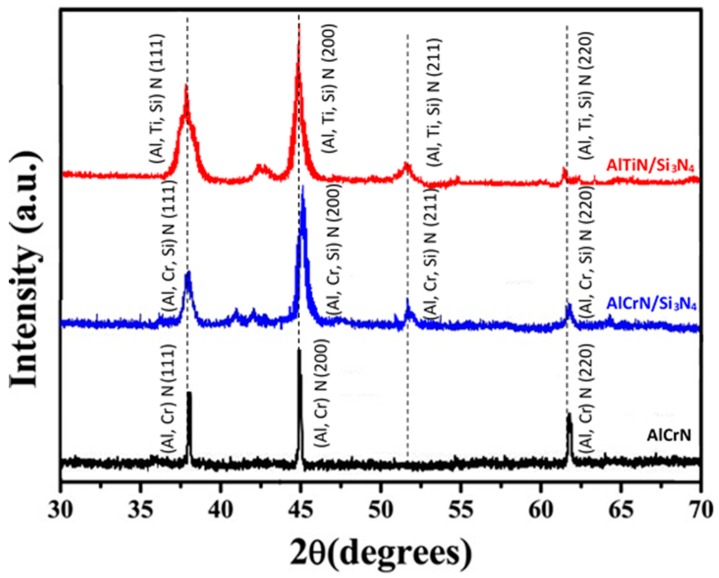
X-ray diffraction (XRD) patterns for coatings deposited under H13.

Figure 4 shows the XRD patterns of the AlCrN and nanocomposite (AlCrN/Si_3_N_4_, AlTiN/Si_3_N_4_) coatings that are deposited over AISI H13 block samples. The patterns clearly show the characteristic peaks of Face Center Cubic (FCC) structure with (111), (200), (220) for all of the coatings.

This FCC metastable solid solution can be obtained in the PVD coating under non-equilibrium conditions of the coating synthesis [17]. In contrast, with the presence of Si for nanocomposite coatings, the diffractograms show that the coatings exhibit a structure with multiple orientations of crystal planes, corresponding to (111), (200), and (220). This result indicates the presence of an amorphous phase of Si_3_N_4_ that is formed during the PVD process deposition on either AlCrSiN or AlTiSiN coatings [17]. Kao et al. [18] using the XRD technique, also provided evidence for the same structure for AlCrSiN and AlTiSiN coating system. According to them, with the presence of Si content, the peaks exhibited a broadening and weakening trend as a whole, which indicated the formation of a fine-grained structure and the decrease of crystalline size; this was due to the incorporation of amorphous Si_3_N_4_ in the coatings. At the same time, the development of the crystal phase was disturbed by the amorphous Si_3_N_4_, causing the nitride grains to grow discontinuously and forming a general nitride mix of aluminum and chromium, or aluminum and titanium, which could effectively affect the property of coating [19,20]. 

### 3.2. Coatings Mechanical Properties

Table 2 is showing the mechanical properties of the coatings studied in this work. The hardness (H) and reduced elastic modulus (E) were determined by nanoindentation. The results show that, with the presence of Si content, both hardness and reduced elastic modulus of coating increase slightly. The highest values for hardness and reduced elastic modulus were reached by AlCrN/Si_3_N_4_ nanocomposite coating. This improvement was also observed for AlTiN/Si_3_N_4_ nanocomposite coatings. The hardness difference of the nanocomposite coatings is related to the different Si contents (Figure 3), which may result from different hardening mechanisms. The presence of Si can lead to the formation of a solid solution hardening (Figure 4), created by the dissolving Si and Al atoms in CrN or TiN (in the case of AlCrN/Si_3_N_4_–AlTiN/Si_3_N_4_) [21,22]. This process will result in a lattice distortion due to having a different atom radius. The amorphous Si_3_N_4_ is thin and envelops CrN or TiN grains, and the interfaces between different phases can hinder dislocation formation or movement, which will lead to a super hard effect. However, it does not mean that a large volume of Si is beneficial for the coatings. For instance, residual stress for AlCrN/Si_3_N_4_ is higher than other coatings that are studied in this work. If the amount of amorphous Si_3_N_4_ matrix increases too much, then the percent of interface area exceeds a certain optimum value, and the phase grain separation and the blocking effect of grain boundaries are limited, which results in high residual stresses [23]. 

**Figure 5:**
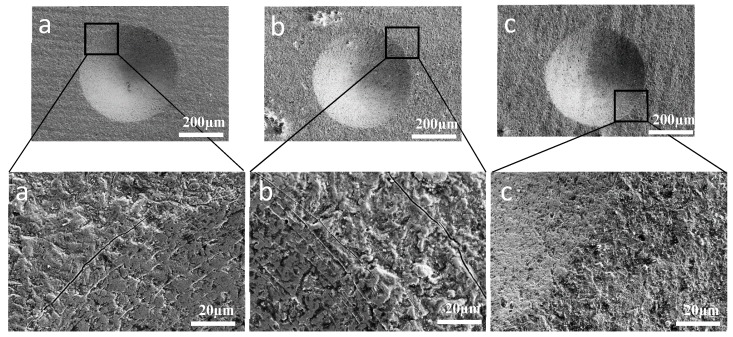
SEM micrographs after Rockwell C indentations with a load of 150 kgf on different coated surfaces (**a**) AlCrN; (**b**) AlTiN/Si_3_N_4_; and (**c**) AlCrN/Si_3_N_4_.

Also, from the H and E values, the toughness of the coatings was evaluated in terms of the relation between H/E and H3/E2 ratios. The values that were calculated are listed in Table 2. This relation results in the ability of a material to resist crack initiation and propagation; the toughness is reflected in the resistance against elastic strain to failure (H/E) and the resistance against plastic deformation (H3/E2). In this case, the H/E and H3/E2 ratios of AlCrN/Si_3_N_4_ were approximately 0.0102 and 0.460 GPa, respectively. This indicates that AlCrN/Si_3_N_4_ coating exhibited the best elastic strain to failure parameter and presents the higher resistance to plastic deformation. This could be confirmed throughout Rockwell adhesion tests done at load of 150 kgf. According to the results presented Figure 5, for all of coatings studied, radial cracks were found around the indentation margin without the presence of peeling. 

Based on VDI guideline 3198 the compound adhesion can be evaluated to adhesion class HF 1 for all samples. This indicates a good adhesion strength of the coatings. However, AlCrN/Si_3_N_4_ presents only very small (almost invisible) cracks around the indentation margin in comparison with other coatings (Figure 5c). This shows that this coating has a better adhesion strength. As it is known, the adhesion strength between the coating and substrate is a critical property in wear-resistance for coatings. If a failure of the coating occurs during operation, then the coating’s capabilities can be greatly reduced, and it can cause severe abrasive wear on the friction system. Therefore, in order to measure this noticeable improvement for the adhesion strength, a scratch test was employed for each sample.

**Figure 6:**
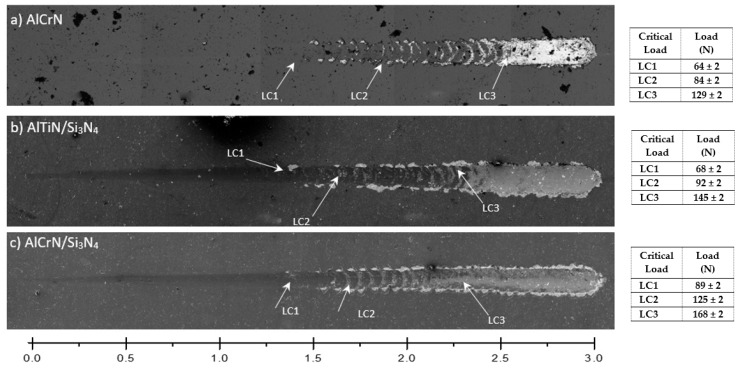
SEM micrographs after scratch test of the coated studied and the Critical load measured at the three stages LC1—First Critical Load (Cohesive Failure); LC2—Second Critical Load (Adhesion Failure) and LC3—Third Critical Load (Substrate Exposure): (**a**) AlCrN; (**b**) AlTiN/Si_3_N_4_; and, (**c**) AlCrN/Si_3_N_4_.

Figure 6 presents the SEM images of scratch tracks for the different coatings that were studied in this paper. From these results, it is possible to see only minor delamination that is found on the scratch track (Figure 6b), demonstrating that AlCrN/Si_3_N_4_ coating has high adhesion strength with the substrate. This can be related to the elastic recovery behavior that takes place in front of the indenter path, which is caused by the compressive stresses that are generated by the indenter and the inability of the coating to deform plastically. This failure mode was observed in LC2 for AlCrN/Si_3_N_4_ with the highest load (125 N), among the three coating system, indicating, therefore, the highest adhesive strength. Obviously, delamination is also formed in the scratch track for AlCrN and AlTiN/Si_3_N_4_ coatings. For these coatings, the load measured in LC2 was 100 N and 92 N, respectively, which proves that the coatings have weaker adhesion strength. Therefore, AlCrN/Si_3_N_4_ is a coating with a higher level of adhesion force, shows a combination of excellent toughness and adhesion strength. These characteristics are essential for improving the die mold service life.

### 3.3. Tribological Properties

**Figure 7:**
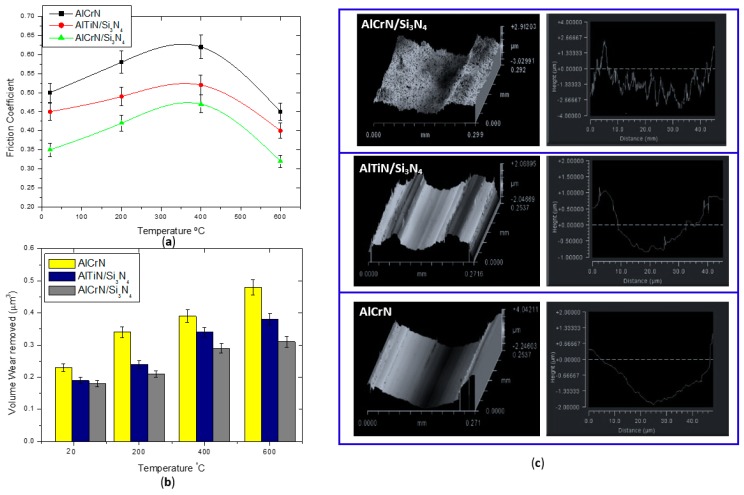
(**a**) Friction coefficient of the coatings at different temperatures; (**b**) Volume wear measured on the top of the surface after pin on disc test and (**c**) three-dimensional (3D) and two-dimensional (2D) profilometer images of the wear track profiles measured on the samples tested at high temperature (600 °C).

The coefficient of friction (COF) vs. temperature data for the coatings in contact with WC ball pin is shown in Figure 7a. 

The coefficient of friction of the AlCrN/Si_3_N_4_ nanocomposite coating is noticeably lower when compared to the other coatings, especially at elevated temperatures within the range of 600 °C. All of the coatings present the characteristics to improve lubricity under elevated temperatures, which can be explained by the presence of oxides that are generated at high temperatures, which tend to reduce the friction conditions at the surface [24]. Also, the COF of both nanocomposite coatings gradually decreases in all stages studied. This behavior is related to the presence of the Si content in the coatings (Figure 3). Similar observation of a decrease in the COF as the Si content increased was also widely reported in other Al-Ti-Si-N based nanocomposite systems [25]. Lower friction might result in longer tool life. To support this hypothesis, soon after the pin-on-disk tests, the overall volume wear that was removed for the coatings was calculated and the results confirm that less friction, in the case of AlCrN/Si_3_N_4_, resulted in much less intensive wear when compared to the other coatings that were studied (Figure 7b). The analysis of the wear volume also revealed a higher wear volume of the AlCrN coating than the nanocomposite coatings for all the different temperatures. Figure 7c shows the corresponding wear tracks at 600 °C for all coatings. The surfaces presented grooves along the sliding direction, indicating that adhesive wear is the prevailing wear mechanism.

**Figure 8:**
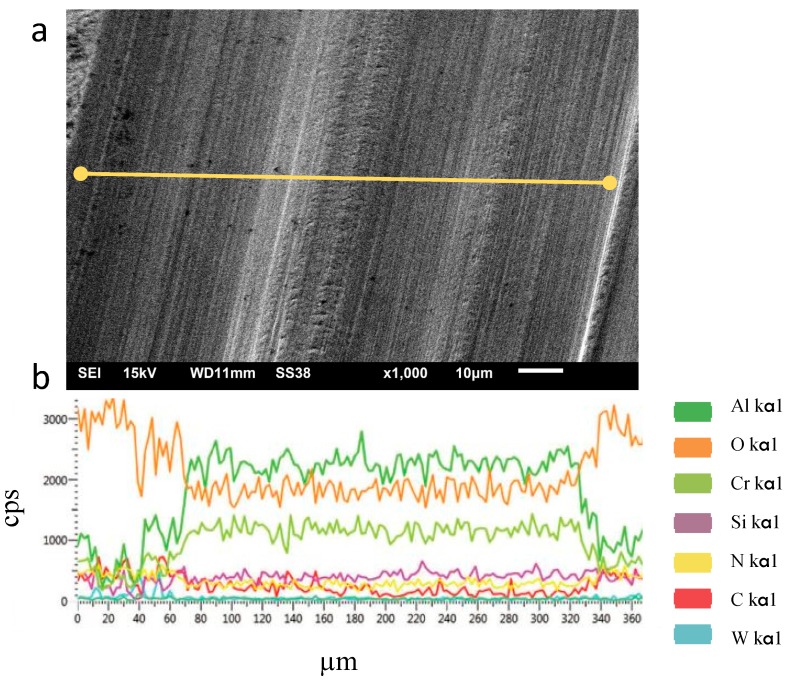
(**a**) EDS spectrum of the drawn line over the track for AlCrN/Si_3_N_4_ coated sample tested at 600 °C and (**b**) spectrum lines.

A way to explain the improvement at frictions conditions for AlCrN/Si_3_N_4_ coating is evaluating the presence of oxides in EDS analysis. This analysis has been done on the wear tracks for AlCrN/Si_3_N_4_ coating. The results that are presented in Figure 8 (a and b) revealed the presence of elements such as Si, Cr, Al, and O, showing a possible formation of the Al_2_O_3_ phase. This oxide is considered a thermal barrier and lubricant layer, and may improve the friction conditions at the work surface. This is a result of the high temperature that is inherent to the process, which causes the appearance of a thin film amorphous structure of the top layer. As shown in Figure 7a, the friction conditions of nanocomposite AlCrN/Si_3_N_4_ tend to reduce at high temperature. Therefore, these results show that, due to the lubricant characteristic and elevated hardness, AlCrN/Si_3_N_4_ nanocomposite coating has excellent wear performance and can contribute to the better wear life of the mold.

### 3.4. Mold’s Tool Life

**Figure 9:**
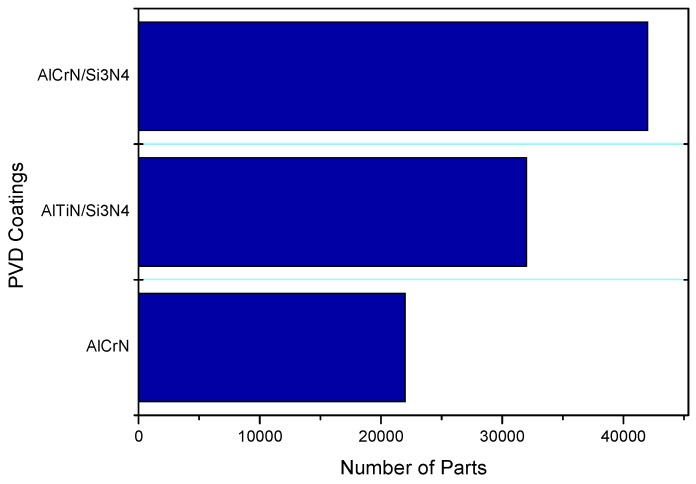
Number of parts produced by high pressure die casting (HPDC) until quality insufficient be achieved.

The behavior of PVD coatings was studied during industrial applications. The tests were done under usual production conditions. For that, the end of the life was determined by obtaining pieces that were free of defects, in other words, the period of time that the machinery could run without stopping for maintenance of the die cast mold. The results of these tests showed that nanocomposite coatings could be used as a novelty in this segment, and that results in an increase of the lifetime significantly in comparison with AlCrN coating (Figure 9). Consequently, the molds coated with AlCrN/Si_3_N_4_ represent a tool life increase of approximately 92% in comparison to the AlCrN coating. This confirms that tool life progress is caused by an improvement of mechanical properties of these coatings (Table 2) within a range of operating temperatures.

**Figure 10:**
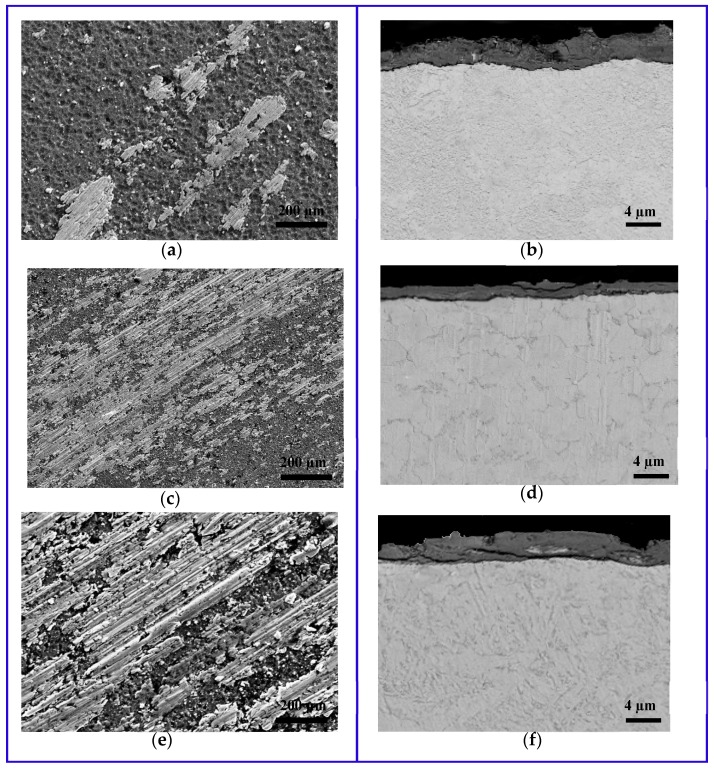
SEM Images of the mold’s surface topography (**a**,**c**,**d**) and core pins cross section (**b**,**d**,**f**) after the HPDC tests. AlCrN/Si_3_N_4_ (**a**,**b**); AlTiN/Si_3_N_4_ (**c**,**d**) and AlCrN (**e**,**f**).

Figure 10 shows SEM images of the top surface of the coated molds and the cross-section of the core pins after the tests. The pins coated with AlCrN suffered severe adhesion and cracking (Figure 10a,b). The microcrack, especially, is caused by the thermal fatigue due to the alternate heating and cooling over the die surface during die casting. Therefore, the die surface tends to be in compression during heating and tension during cooling of the die. This results in thermal cracking on the die surface, which degrades part of the surface finish and ultimately leads to die failure. For the nanocomposite coatings, the main wear problems during continuous die-casting were not microcracks, but erosion and sticking of the molten aluminum due to the regular flow during the casting process. For AlTiN/Si_3_N_4_, areas of the coating were detached by erosion (Figure 10c,d). Adhesion and erosion phenomena were particularly reduced for the AlCrN/Si_3_N_4_ (Figure 10e,f).

### 3.5. Results Outline

The use of conventional coatings, such as TiN, CrN, (Cr,Al)N, employed in aluminum die cast molds has been showed a limited performance during operation at temperatures range of 400 °C to 600 °C. Requirements of higher operating temperatures up to 1200 °C have led to the development of more complex nanocomposite nitride coatings such as AlTiN and AlCrSiN. Nanocomposite coatings consist of a hard crystalline phase (e.g., AlTiN, AlCrN grains) that is embedded in an amorphous matrix (a-Si_3_N_4_, a-C) with Si being present either in the form of a solid solution or as a separate a-Si_3_N_4_ matrix/phase in which nanocrystalline AlTiN or AlCrN phase is embedded, forming consequently nc-AlTiN/a-Si_3_N_4_ or nc-AlCrN/a-Si_3_N_4_. Due to their unique nanostructural design, nanocomposite coatings exhibit increased hardness levels of >40 GPa and strongly improved thermal stability at temperatures of up to 1200 °C [26,27]. Therefore, the wear resistance during their operation is known to be governed by the composition of some functional and mechanical properties for instance adhesion, hardness, hot hardness, residual stress level, and other mechanical properties.

In this way, it was shown experimentally that nano-composite coatings, in particular, AlCrN/Si_3_N_4_ coating demonstrated the best performance. This is an optimum combination of hardness, stress state adhesion, oxidation resistance and soldering behavior. The higher adhesion resistance of AlCrN/Si_3_N_4_ is a result of the combination of the microstructure of the CrAlSiN coatings, which was composed of (Cr,Al)N crystallites combined with the amorphous Si_3_N_4_ phase [28]. It leads to the better performance during the die casting process.

This coating also exhibited low friction characteristics at 600 °C in relation the other coatings studied. The high hardness of nanocomposite coatings at elevated temperature leads to a reduction of seizure intensity at the tool/workpiece interface that is caused by plastic deformation of the surface layers. The application of AlCrN/Si_3_N_4_ also leads to an increase in the lifetime of aluminum die-casting dies through reduction of erosion, corrosion, and soldering processes, and due to an increase of the thermal fatigue limit. Moreover, the molds are generally subjected to a series of operational stresses during die casting. These are mainly thermal stresses, resulting from thermal cycling of the material each time the mold is filled. Based on the results that were obtained in this work, we propose that the nanocomposite coatings that are deposited by LERC technology are very well suited for large and heavy dies with a complex shape, providing a uniform coating thickness and homogeneity.

## 4. Conclusions

AlCrN/Si_3_N_4_ nanocomposite PVD coating showed an optimum combination of hardness, adhesion, soldering behavior, oxidation resistance, and stress state. These characteristics are essential for improving the die mold service life. 

Due to the presence of nanocrystals of AlCrN dispersed in an amorphous matrix of Si_3_N_4_, this coating showed the ability to resist severe mechanical and thermal stress conditions. It provides an efficient stress barrier, preventing crack propagation that is caused by dynamic pressing. As a final result, the application of nano-composite coating increases the lifespan by approximately three times as compared to the benchmark AlCrN coating. The surface damage due to wear for this coating was minimal.

Therefore, according to the goal of this paper and considering the experimental results, nano-composite AlCrN/Si_3_N_4_ PVD coating deposited by pulsed cathodic arc evaporation equipped with lateral and central arc-rotating cathodes has been proposed as a suitable novel solution for surface engineering of aluminum die casting molds. The results showed that this coating exhibited advantages in terms of wear and tribological performance.

## Figures and Tables

**Table:** 

Coating	(a)—AlCrN	(b)—AlTiN/Si_3_N_4_	(c)—AlCrN/Si_3_N_4_
Element	Atomic %	Atomic %	Atomic %
Al K	31.6	20.5	27.3
Cr K	18.1	-	22.7
Ti K	-	16	-
Si K	-	3.5	3.9
N K	50.3	60	51.1

**Table 1:** Chemical composition of AISI H13 used as die cast mold substrate.

Element/Amount	C	Cr	Mo	Mn	Si	V	Fe
wt %	0.49	4.99	1.3	0.4	0.97	0.93	Balance

**Table 2:** Mechanical properties, residual stress, and thickness of the coatings.

Coatings	H—Hardness (GPa)	E—Reduced Elastic Modulus (GPa)	H/E Ratio	H3/E2 Ratio	Residual Stress (GPa)	Thickness (µm)
AlCrN	27 ± 3	360 ± 25	0.0750	0.151	4	3.2
AlTiN/Si_3_N_4_	39 ± 3	400 ± 25	0.0975	0.370	4.8	2.8
AlCrN/Si_3_N_4_	44 ± 3	430 ± 25	0.0102	0.460	5.2	2.3

**Table 3:** The surface roughness of the coatings.

Coatings	Surface Roughness—Ra (µm)
Uncoated	0.88 ± 0.02
AlCrN	1.59 ± 0.02
AlTiN/Si_3_N_4_	1.43 ± 0.02
AlCrN/Si_3_N_4_	1.38 ± 0.02
